# The role of the maximum involvement of biopsy core in predicting outcome for patients treated with dose-escalated radiation therapy for prostate cancer

**DOI:** 10.1186/1748-717X-7-127

**Published:** 2012-08-01

**Authors:** Jure Murgic, Matthew H Stenmark, Schuyler Halverson, Kevin Blas, Felix Y Feng, Daniel A Hamstra

**Affiliations:** 1 The Department of Radiation Oncology, University of Michigan Medical Center, Ann Arbor, MI, USA; 2 Veterans Affairs Medical Center, Ann Arbor, MI, USA; 3 University of Michigan Health System, Department of Radiation Oncology, 1500 E. Medical Center Drive, UH B2-C490, Ann Arbor, MI, 48109-5010, USA

**Keywords:** Prostate cancer, Biopsy, prognostic factors, Maximum involvement, Tumor in Core, Radiotherapy

## Abstract

**Purpose:**

To evaluate the influence of the maximum involvement of biopsy core (MIBC) on outcome for prostate cancer patients treated with dose-escalated external beam radiotherapy (EBRT).

**Methods and materials:**

The outcomes of 590 men with localized prostate cancer treated with EBRT (≥75 Gy) at a single institution were retrospectively analyzed. The influence of MIBC on freedom from biochemical failure (FFBF), freedom from metastasis (FFM), cause-specific survival (CSS), and overall survival (OS) was compared to other surrogates for biopsy tumor volume, including the percentage of positive biopsy cores (PPC) and the total percentage of cancer volume (PCV).

**Results:**

MIBC correlated with PSA, T-stage, Gleason score, NCCN risk group, PPC, PCV, and treatment related factors. On univariate analysis, MIBC was prognostic for all endpoints except OS; with greatest impact in those with Gleason scores of 8–10. However, on multivariate analysis, MIBC was only prognostic for FFBF (hazard ratio [HR] 1.9, *p* = 0.008), but not for FFM (*p* = 0.19), CSS (*p* = 0.16), and OS (*p* = 0.99).

**Conclusions:**

In patients undergoing dose-escalated EBRT, MIBC had the greatest influence in those with Gleason scores of 8–10 but provided no additional prognostic data as compared to PPC and PCV, which remain the preferable prognostic variables in this patient population.

## Introduction

Pretreatment prognostic indices predictive of outcome in patients with clinically localized prostate cancer typically rely on risk-factors including: prostate-specific antigen (PSA), clinical T-stage, and biopsy Gleason score (GS) [1,2]. More recent models have evaluated the prognostic utility of incorporating biopsy tumor volume. Surrogates for cancer volume have included the percentage of positive cores (PPC) at the time of prostate biopsy [3,6] and the total percentage of cancer volume (PCV) in all of the biopsy cores [7,8]. In a recent analysis we demonstrated that in a cohort of patients treated with dose-escalated EBRT, PCV was superior to PPC as a prognostic variable for prediction of clinical outcomes. In addition, PCV was found to add prognostic significance for all end-points, including overall survival (OS) [8]. However, calculating PCV is time consuming; furthermore, it is possible that a moderate volume of cancer involved in a large number of cores is less important than a dominant lesion involving a large volume of cancer in one or more cores. The maximum involvement of a single biopsy core has previously been demonstrated to correlate with worse pathologic features and higher biochemical failure following radical prostatectomy; however, it has not previously been assessed in patients treated with EBRT. Therefore, this study aimed to assess the prognostic significance of the maximum involvement of biopsy core by cancer (MIBC) as compared to both PPC and PCV in a cohort of patients treated with dose-escalated EBRT for prostate cancer.

## Patients and methods

### Patients

From 1998 to 2008, 718 men with clinically localized prostate adenocarcinoma were consecutively treated at the University of Michigan with definitive external beam radiotherapy to a minimum dose of 75 Gy with or without neo-adjuvant and/or adjuvant androgen deprivation therapy (ADT). Prior to treatment, patients were risk stratified into low-, intermediate-, and high-risk groups based on standard NCCN criteria. Staging evaluation was performed per standard clinical practice. Symptomatic patients and/or those with NCCN-defined high-risk features were routinely staged with pelvic CT and bone-scan. Patients with evidence of metastatic disease were excluded from the analysis.

All prostate core biopsies were reviewed by dedicated uropathologists at the University of Michigan who reported Gleason score, number of positive cores, and percent cancer involvement for each core. The maximum involvement of biopsy core (MIBC) was defined as the highest percentage of cancer present in one or more individual biopsy cores from all sampled cores. Percentage of positive cores (PPC) and the percentage cancer volume (PCV) were both calculated as previously described [6,8].

### Treatment

All patients underwent CT-based treatment planning and were treated with 3D-conformal RT (3D-CRT) or intensity modulated RT (IMRT). The median prescribed dose was 78 Gy (inter-quartile range [IQR] 76–78) using daily fractions of 1.8 to 2.0 Gy. Treatments were planned to ensure 95% coverage of the planning target volume (PTV) by the prescription isodose level. Clinical target volumes (CTV) were typically based upon the NCCN risk-stratification criteria such that low-risk patients were treated to the prostate only, intermediate-risk patients to the prostate and seminal vesicles, and high-risk patients to the pelvic lymph nodes to 45 Gy followed by a boost to the prostate and seminal vesicles. The frequency and duration of ADT were as follows: low-risk (11%, median 4.1 months), intermediate-risk (27%, median 6.6 months), and high-risk (90%, median 23.0 months).

### Outcome assessments

Patients were routinely followed at 3–6 month intervals for the first 5 years and every 6 to 12 months thereafter. Freedom from biochemical failure (FFBF) was defined based upon the ASTRO Phoenix definition (20). Freedom from metastasis (FFM) was defined as the absence of any clinical, radiographic, or pathologic evidence of metastatic disease. Cause specific survival (CSS) was defined as death attributed to prostate cancer or death in any patient with either castrate-resistant prostate cancer or evidence of metastatic disease prior to death. OS was defined as death due to any cause.

### Statistical methods

One-way analysis of variance (ANOVA) was used for the comparison between MIBC groups and continuous variables while the chi-square (CS) test was used for analysis between MIBC groups and categorical variables. Univariate survival analyses were conducted using the log-rank test and Kaplan-Meier method. Multivariate analyses were conducted using Cox proportional hazards models. Receiver operator characteristic (ROC) curves were used to evaluate the relative predictive capacity of MIBC compared to other cancer volume metrics (PPC, PCV), used as continuous variables and correlated with clinical end-points at 7 years. The cut-point that best discriminated patient outcome based on different metrics was computed using the maximum likelihood ratio. All statistical analysis was performed using MedCalc (v11.4.4.0, MedCalc Software, Mariakerke, Belgium) using 2-sided tests with a p-value <0.05 considered significant.

## Results

### Clinical characteristics

Data on MIBC, PPC, and PCV was available for 590 of 718 (82%) men with a median age of 69 years (IQR: 63–74) and a median follow-up of 57 months (IQR: 34–81). The median number of biopsy cores sampled was 8 (IQR: 6–12, range: 4–86) with a median PPC of 33.3% (IQR: 17-60%; range: 3.7-100%), a median PCV of 10% (IQR: 2.5-25%; range: 0.14-95%), and a median MIBC of 30.0% (IQR: 10-70%, range 1-100%).

The relationship between MIBC and other clinical and tumor related characteristics is shown in Table 1. MIBC was associated with NCCN risk group as well as individual risk features including PSA, T-Stage, and Gleason score (all *p*-values <0.001) with increasing MIBC quartiles associated with more advanced clinical features. When divided by NCCN risk-group, the proportion of patients with low-, intermediate-, and high-risk disease within the 1^st^ MIBC quartile was 52%, 35% and 13%, respectively, while within the 4^th^ MIBC quartile it was 5%, 39%, and 56%. There were also differences in the use of pelvic RT and ADT between MIBC subgroups, such that only 12% and 29% in 1^st^ MIBC quartile received pelvic RT and ADT, respectively, while 55% and 66% of patients in 4^th^ quartile received these treatments.

**Table 1:** Clinical and treatment characteristics for maximum involvement of biopsy core (MIBC) stratified by quartile

**Variable**	**MIBC by quartile**				
	**1**^**st**^**(<10%)**	**2**^**nd**^**(<30%)**	**3**^**rd**^**(<70%)**	**4**^**th**^**(≥70%)**	***p***
Patients (*n*)	144	135	147	164	
Age (*y*), median (IQR)	68 (62–73)	69 (63–74)	69 (62–74)	71 (64–75)	0.48*
PSA (ng/mL), median (IQR)	7.6 (5.3-10.6)	7.2 (4.9-10.4)	7.5 (5.0-12.2)	10.4 (6.7-20.4)	<0.001*
<10	70%	73%	65%	48%	<0.0001^†^
10-20	23%	17%	25%	27%	
≥20	7%	10%	10%	26%	
T-stage					<0.0001^†^
T1-T2a	91%	92%	79%	59%	
T2b-T2c	6%	4%	17%	26%	
T3-T4	3%	4%	4%	15%	
Gleason score					<0.0001^†^
2-6	67%	54%	22%	8%	
7	26%	39%	60%	49%	
8	5%	6%	10%	17%	
9-10	2%	1%	8%	26%	
Percent cores positive	17% (13-33%)	25% (17-40%)	41% (25-58%)	63% (46-83%)	<0.001*
Percent cancer volume	0.8% (0.5-1.7%)	2.9% (1.9-5.0%)	11.7% (6.7-18.8%)	33.6% (20.8-48.9%)	<0.001*
NCCN risk group					<0.0001^†^
Low	52%	37%	14%	5%	
Intermediate	35%	45%	59%	39%	
High	13%	18%	27%	56%	
RT dose (Gy), median (IQR)	77 (76–78)	77 (76–78)	77 (77–78)	78 (77–78)	<0.001*
Pelvic RT	12%	17%	25%	55%	<0.0001^†^
ADT use	29%	31%	35%	66%	<0.0001^†^
ADT duration, median (IQR)	6.9 (6.0-18.7)	6.1 (4.0-18.7)	6.4 (6.0-24.4)	20.9 (6.3-25.6)	0.2^*^

Abbreviations: IQR = inter-quartile range; PSA = prostate-specific antigen; NCCN = National Comprehensive Cancer Network; RT = radiotherapy; ADT = androgen deprivation therapy.

*Analysis of variance.

^†^Chi-square test.

**Figure 1:**
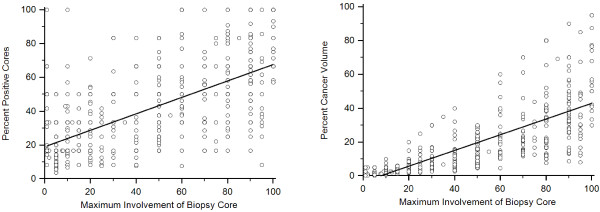
(a) Correlation between maximum involvement of biopsy core (MIBC) and (a) percentage of positive cores (PPC) and (b) percentage of cancer volume (PCV).

Both PCV and PPC were significantly correlated with MIBC (*p* <0.001) with a much weaker correlation between MIBC and PPC (r = 0.52, 95%CI: 0.45-0.57, Figure 1A) as compared to MIBC and PCV (r = 0.77, 95%CI: 0.73-0.80, Figure 1B).

### Association between MIBC and clinical outcome

When analyzed by quartile, MIBC demonstrated significant correlation with FFBF (*p* < 0.0001), FFM (*p* < 0.005), and CSS (*p* < 0.007) and borderline association with OS (*p* = 0.06), (Table 2). For all end-points, the 4^th^ quartile (70%) exhibited significantly worse clinical behavior than the lower three quartiles. When the 4th quartile was excluded, there was only a difference in FFBF (*p* < 0.009) across the first three quartiles but no difference in FFM (*p* = 0.12), CSS (*p* = 0.29), or OS (*p* = 0.30) (Table 2). Since ADT use was highly correlated with increasing risk-features there was also a close correlation between increasing MIBC and ADT use (No ADT: MIBC median 20 (IQR:5–50); with ADT: MIBC median 60 (10–95), ANOVA p < 0.001). After dividing the data by ADT use MIBC was prognostic only for BF (p = 0.02) and metastasis (p = 0.03) in those treated with RT alone and was only prognostic for BF in those treated with RT plus ADT (p = 0.01).

**Table 2:** Univariate Analysis of Clinical Outcome As A Function of MIBC By Quartile

**Quartile**	**N**	**FFBF**		**FFM**		**CSS**		**OS**	
		**5 yr**	**8 yr**	**5 yr**	**8 yr**	**5 yr**	**8 yr**	**5 yr**	**8 yr**
<10%	144	91%	91%	97%	95%	100%	96%	91%	72%
		(88–94)	(88–94)	(95–99)	(92–98)		(93–99)	(88–94)	(55–79)
<30%	134	93%	79%	97%	93%	100%	100%	96%	92%
		(90–96)	(72–86)	(95–99)	(88–98)			(94–98)	(89–95)
<70%	147	82%	70%	95%	83%	99%	99%	95%	82%
		(88–86)	(63–77)	(93–97)	(77–89)	(98–100)	(98–100)	(93–97)	(77–87)
70 +%	164	74%	51%	91%	82%	94%	94%	87%	77%
		(71–78)	(43–59)	(88–94)	(77–87)	(92–96)	(92–96)	(84–90)	(72–82)
p-value Overall		<0.0001		0.0047		0.0071		0.061	
p-value Quartiles 1-3		0.009		0.12		0.29		0.30	

Rates at five and eight years along with the standard error of the mean.

To identify the optimal cut-point for MIBC stratification, ROC curves were generated for each endpoint using MIBC as a continuous variable. At 7-years, MIBC was predictive for FFBF (area under the curve [AUC]: 0.67, 95% CI: 0.60-0.74, *p* < 0.0001), FFM (AUC: 0.67, 95% CI: 0.58-0.75, *p* = 0.004), and CSS (AUC: 0.79, 95% CI: 0.69-0.87, *p* = 0.0002), but not OS (AUC: 0.60, 95% CI: 0.51-0.69, *p* = 0.075). A number of different cut-points could be utilized for further analysis and indeed given close association between increasing risk-features and increasing MIBC if MIBC was addressed in 10% increments any cut-point >10% was associated with BF while any cut-point >40% was associated with metastasis and death from prostate cancer. From these analyses MIBC had the strongest prognostic association with death from prostate cancer (AUC 0.79) and a cut-point of 60% was selected for further evaluation as this value was most closely associated with CSS, (negative predictive value [NPV] 97% and positive predictive value of 30.5%) while still maintaining modest prognostic significance for FFBF (NPV 64%) and FFM (NPV 87%).

**Figure 2:**
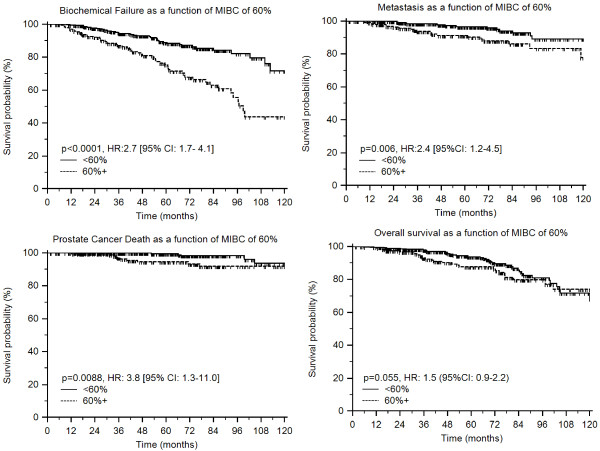
** Kaplan-Meier estimates of (a) freedom from biochemical failure, (b) freedom from metastasis, (c) cause-specific survival, and (d) overall survival as a function of maximum involvement of biopsy core (MIBC).** Cut-point of 60% generated from receiver operating characteristic curve analysis.

On univariate analysis, those with MIBC of 60% or greater (n = 196) had worse clinical outcome than those with MIBC of less than 60% (n = 394). Stratification according to this MIBC cut-point of 60% was prognostic for FFBF (*p* < 0.0001, HR:2.7 [95% CI: 1.7- 4.1]), FFM (*p* = 0.006, HR:2.4 [95%CI: 1.2-4.5]), and CSS [p = 0.0088, HR: 3.8 [95% CI: 1.3-11.0]) with borderline association with OS (p = 0.055, HR: 1.5 [95%CI: 0.9-2.2]) (Figure 2A-D).

### Multivariate analysis

Given the correlation between MIBC and conventional clinical risk-groups, multivariate Cox-proportional hazards modeling was performed stratifying patients by NCCN risk-grouping and the best-identified cut-point for MIBC (60%). The presence of high-risk disease was the strongest predictor of decreased FFBF, FFM, CSS, and OS with hazard ratios (HR) ranging from 3.0 to 6.9 (Table 3). Conversely, after including MIBC intermediate-risk disease was not prognostic for any of these endpoints. However, after adjusting for NCCN risk-groups, a large volume of cancer in any one core (as defined by MIBC >60%) provided further prognostic significance for FFBF (p = 0.008, HR:1.9 [95% CI: 1.2-2.9]) but did not influence any other end-points. In an additional multivariate analysis, MIBC was analyzed as continuous variable and after controlling for risk-group, each 10% increment in MIBC yielded an increased risk for biochemical failure (p = 0.0008, HR: 1.13 [95% CI: 1.1-1.2]) with no impact upon other clinical end-points.

**Table 3:** Multivariate analysis for all clinical outcomes stratified for NCCN risk group and the best-identified cut-point for MIBC (60%)

**Covariate**	**FFBF**		**FFM**		**CSS**		**OS**	
	**HR (95% CI)**	***p***	**HR (95% CI)**	***p***	**HR (95% CI)**	***p***	**HR (95% CI)**	***p***
NCCN risk group								
Low	Reference		Reference		Reference		Reference	
Intermediate	1.7 (0.83-3.4)	0.15	2.8 (0.80-9.8)	0.11	2.1 (0.23-19.7)	0.51	1.2 (0.57-2.5)	0.65
High	3.2 (1.6-6.5)	0.001	4.6 (1.3-16.2)	0.02	6.9 (0.8-59.6)	0.08	3.0 (1.5-6.1)	0.002
MIBC								
<60%	Reference		Reference		Reference		Reference	
≥60%	1.9 (1.2-2.9)	0.008	1.6 (0.81-3.1)	0.19	2.2 (0.72-6.9)	0.16	1.0 (0.59-1.7)	0.99

*Abbreviations*: MIBC = maximum involvement of biopsy core; FFBF = freedom from biochemical failure; FFM = freedom from metastasis; CSS = cause specific survival; OS = overall survival; HR = hazard ratio; CI = confidence interval.

To further evaluate the influence of MIBC as compared to PPC and PCV univariate analysis and multivariate analysis was performed for BF using relevant pre-treatment and treatment-related features (Table 4). When only MIBC was included in a multivariate model (either as a continuous variable [presented] or by 60% cut-point [not presented]) MIBC did add prognostic value for BF (Model #1). Each 1% increase in MIBC resulted in a 1% increase in the relative risk of biochemical failure even after controlling for other clinical features. However, once either PPC (Model #2) or PCV (Model #3) were introduced MIBC no longer retained prognostic significance while both PPC and PCV did. Similar analyses were performed for metastasis, CSS, and OS and in each case MIBC did not add prognostic value, and so they were not presented.

**Table 4:** Univariate and Multivariate Models For Freedom From Biochemical Failure

**Variable**	**Univariate Analysis**		**Multivariate Model # 1 Maximum Core Only**		**Multivariate Model #2 MIBC + Percent Positive Cores**		**Multivariate Model #3 MIBC + Percent Cancer Volume**	
	**p-value**	**Hazard Ratio (95% CI)**	**p-value**	**Hazard Ratio (95%CI)**	**p-value**	**Hazard Ratio (95%CI)**	**p-value**	**Hazard Ratio (95%CI)**
Age	0.62	0.99 (0.97-1.02)	0.23	0.99 (0.96-1.01)	0.40	0.99 (0.97-1.01)	0.48	0.99 (0.97-1.02)
PSA	<0.0001	5.7 (3.6-9.5)	<0.0001	4.6 (2.3-9.3)	<0.0001	4.7 (2.3-9.7)	0.0001	4.5 (2.2-9.4)
T-stage								
T1-T2a	Reference		Reference		Reference		Reference	
T2b-T2c	0.0065	2.0 (1.2-3.3)	0.48	1.2 (0.70-2.2)	0.82	1.1 (0.59-1.9)	1.0	1.00 (0.55-1.8)
T3-4	<0.0001	3.3 (1.9-5.6)	0.67	1.2 (0.60-2.2)	0.83	1.1 (0.55-2.1)	0.91	0.96 (0.49-1.9)
Gleason Score								
2-6	Reference		Reference		Reference		Reference	
7	0.0004	2.6 (1.5-4.3)	0.17	1.6 (0.82-3.1)	0.45	1.3 (0.67-2.5)	0.58	1.2 (0.61-2.4)
8-10	<0.0001	4.9 (2.8-8.9)	0.02	2.8 (1.2-6.9)	0.027	2.8 (1.1-6.7)	0.07	2.3 (0.93-5.8)
ADT Use								
No	Reference		Reference		Reference		Reference	
Yes	0.0001	2.2 (1.5-3.3)	0.82	0.94 (0.53-1.7)	0.68	0.88 (0.49-1.6)	0.80	0.93 (0.51-1.7)
Pelvic RT								
No	Reference		Reference		Reference		Reference	
Yes	<0.0001	2.7 (2.0-3.8)	0.32	0.68 (0.32-1.5)	0.20	0.59 (0.27-1.3)	0.30	0.65 (0.29-1.5)
RT Dose	0.10	1.2 (0.97-1.4)	0.95	1.01 (0.78-1.3)	0.71	1.05 (0.82-1.3)	0.70	1.05 (0.81-1.4)
Cancer Volume Metrics								
Maximum Biopsy Core	<0.0001	1.02 (1.01-1.02)	0.12	1.01 (1.00-1.02)	0.27	1.00 (0.99-1.02)	0.33	1.00 (1.0-1.02)
Percent Positive Cores	<0.0001	1.02 (1.01-1.03)			0.019	1.01 (1.00-1.02)		
Percent Cancer Volume	<0.0001	1.02 (1.02-1.03)					0.03	1.02 (0.98-1.04)

All analyses done by Cox Proportional Hazards regression with variables modeled as continuous variables except where indicated.

*Abbreviations*: MIBC = maximum involvement of biopsy core; PSA = Prostate Specific Antigen; ADT = Androgen Deprivation Therapy; RT = Radiation Therapy; HR = hazard ratio; CI = confidence interval.

**Figure 3:**
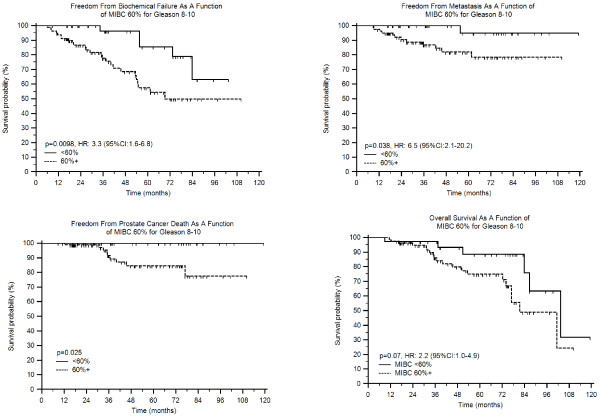
** Failure patterns for patient cohort based on Gleason 8–10 stratified by having less than or equal to 60% MIBC vs. greater than 60% MIBC.** (**a**) FFBF. (**b**) FFM. (**c**) CSS (**d**) OS.

Given the close association between Gleason score and MIBC which was 10% (IQR:5-25%), 40% (IQR:20-70%) and 80% (IQR:50-90%) for Gleason 2–6, 7, and 8–10, respectively (p < 0.001, ANOVA) we assessed whether MIBC had differing prognostic significance as a function of Gleason score. MIBC had no prognostic impact in those with Gleason 2–6 or 7 for any clinical end-point assessed (all p > 0.1); however, if Gleason was combined in those with scores of 2–7 the MIBC did predict worse BF using a cut-point of 60% (p = 0.0036, HR: 2.0 (95%CI:1.2-3.4). Nevertheless, even in this combined Gleason 2–7 group (n = 474) MIBC did not influence metastasis, CSS, or OS (all p-values >0.1, data not shown). However, in the 116 patients with Gleason 8–10 MIBC clearly influenced clinical outcome (Figures 3A-3D) with worse BF (p = 0.0098, HR:3.3 (95%CI:1.6-6.8), metastasis (p = 0.038, HR:6.5 (95%CI:2.1-20)), CSS (p = 0.025), and borderline for OS (p = 0.073, HR:2.2 (95%CI:1.0-4.9)). Interestingly, in those with Gleason 8–10 and MIBC >60% (n = 37, median follow-up 57 months) there were no deaths from PCa while in those with Gleason 8–10 and MIBC<60% (n = 79, median follow-up 39 months) there were 9 deaths from PCa (Figure 3C). (Since there were no deaths in those with MIBC<60% and Gleason scores of 8-10 a hazard ratio could not be determined).

Finally, we evaluated the hypothesis that MIBC would have greater prognostic significance in patients with a low total volume of cancer where the impact of a single high volume core might be more noticeable. Within this data set, we previously identified an optimal cut-point of 22.5% for total biopsy cancer volume (PCV <22.5% *n* = 393]; PCV ≥22.5% *n* = 144]), which on multivariate analysis predicted worse outcome for all clinical endpoints, including CSS (HR 3.9, *p* = 0.01) [8]. Of the 144 patients with a large total cancer volume, MIBC (stratified by 60%) was not prognostic for any end-point (p > 0.3 for all). However, in the 393 patients with a smaller total volume of cancer, 67 patients (17%) had maximum core involvement of ≥60% with a trend toward worse FFBF (HR: 1.7 [95%CI: 0.8-3.9], *p* = 0.09) and FFM (HR: 2.2 [95%CI:0.7-6.7], *p* = 0.07) but no impact upon CSS or OS (*p* > 0.5 for each).

## Discussion

One limitation to predicting outcome in men undergoing radiotherapy for prostate cancer is the poor ability to define the volume of cancer present at the time of diagnosis. Both PPC and PCV have previously been evaluated as prognostic tools for patients treated with radiation therapy [3-8]. In contrast, data on the prognostic impact of MIBC in prostate cancer are scarce; to our knowledge this is the first analysis on the utility of MIBC in patients treated with EBRT.

In one previous report MIBC was identified as an independent determinant of histological disease progression in a cohort of patients with low-risk prostate cancer undergoing active surveillance [9]. Moreover, in a comprehensive analysis done by Brimo et al., the authors tested the prognostic value of seven different morphometric measurements of tumor extent from prostate needle core biopsy tissue, including MIBC, in a group of patients treated with radical prostatectomy. On univariate analysis the time to PSA recurrence was marginally associated with greatest percentage of cancer (p = 0.06) which was defined in a similar manner to how we defined MIBC [10].

In the present study, MIBC was closely correlated with other clinical risk-features as well as PCV (r = 0.77) and less so with PPC (r = 0.52). Given the close association with clinical risk-features, it is not surprising that on univariate analysis MIBC was associated with clinical outcome for all end-points except OS. However, on multivariate analysis either after controlling for NCCN risk-group or for other clinical and treatment-related features, MIBC only added prognostic value for FFBF, but not for any of the other clinically relevant end-points. Despite limited prognostic significance over all patients; when MIBC was assessed independently in patients with Gleason scores of 8–10 it did correlate significantly with greater BF, metastasis, and death from prostate cancer with borderline association with OS while in those with Gleason scores of 2–7 it only appeared to influence BF.

Conversely, within the same dataset, PCV predicted a worse outcome for all end-points on multivariate analysis, including FFBF (HR 1.9, *p* = .003), FFM (HR 1.7, *p* = .09), CSS (HR 3.9, *p* = .01), and OS (HR 1.8, *p* = .02) even after accounting for PPC.[8] Thus, at present, PPC and PCV should remain the standard metrics for estimating prostate cancer volume, given the additional prognostic data they provide as compared to MIBC for patients undergoing definitive EBRT. Interestingly, as previously observed when any of the measures of cancer volume (MIBC, PPC, or PCV) were included in multivariate models clinical T-stage was no longer prognostic which is suggestive that these measures of cancer volume are likely more clinically relevant than T-stage assessed by digital rectal exam.[8,11] The strengths of study include the large patient number, restriction to patients undergoing dose-escalated EBRT, and analysis of clinically relevant endpoints, including FFM, CSS, and OS. Limitations arise from the study are significant given that it represents retrospective analysis from a single institutions without central pathologic review or validation of these findings. In addition, the correlation between MIBC and both the use of pelvic RT and ADT certainly cloud the ability to independently evaluate the impact of each of these factors while the follow-up of only 5 years limits conclusions about more long-term end-points such as metastasis and CSS.

## Conclusion

Although a simple and an easily obtained measure of prostate cancer volume, MIBC provided no additional prognostic data compared to PPC and PCV, and indeed was less relevant then either of these other metrics. Therefore, both PPC and PCV are preferable end-points to be used as prognostic tools. However, if future analyses of MIBC are undertaken we would suggest a focus upon patients with the highest Gleason scores where MIBC had the greatest suggestion of possible value.

## Abbreviations

MIBC: Maximum involvement of biopsy core; PPC: percentage of positive biopsy cores; PCV: total percentage of cancer volume; EBRT: external beam radiation therapy; FFBF: freedom from biochemical failure; FFM: freedom from metastasis; CSS: cause-specific survival; OS: overall survival; PSA: prostate specific antigen; NCCN: national comprehensive cancer network; HR: hazard ratio; IQR: inter-quartile range; ADT: androgen deprivation therapy; 3D-CRT: three-dimensional conformal radiation therapy; IMRT: intensity modulated radiation therapy; RT: radiation therapy; ANOVA: one-way analysis of variance; CS: chi-square; ROC: receiver operator characteristic; AUC: area under the curve.

## Competing interest

The authors report no financial conflicts of interest.

## Authors’ contributions

JM was responsible for study concept, data analysis, and drafting the manuscript. MS was responsible for study concept, data analysis, and drafting the manuscript. SH and KB were responsible for study design, data collection, and drafting the manuscript. FF and DH were responsible for study design, data collection, data analysis, drafting the manuscript, and supervision of the other authors. DH was responsible for final manuscript preparation. All authors read and approved the final manuscript.
